# Oral Cancer Awareness, Attitudes, and Barriers among Jordanian Adults: A Cross-sectional Study

**DOI:** 10.3290/j.ohpd.b2805373

**Published:** 2022-03-14

**Authors:** Fadi S. Jarab, Walid Al-Qerem, Raghda Qarqaz

**Affiliations:** a Assistant Professor, Department of Oral Medicine and Surgery, Faculty of Dentistry, Jordan University of Science and Technology, Irbid, Jordan. Conceptualisation, investigation, methodology, supervision, project administration, validation, manuscript writing, review and editing.; b Associate Professor, Department of Pharmacy, Al-Zaytoonah University of Jordan, Amman, Jordan. Formal analysis, investigation, methodology, supervision, manuscript writing, review and editing.; c Research Assistant, Department of Pharmacy, Al-Zaytoonah University of Jordan, Amman, Jordan. Data curation, formal analysis, wrote original draft.

**Keywords:** attitudes, barriers, Jordan, knowledge, oral cancer, screening

## Abstract

**Purpose:**

To identify the gaps in the Jordanian population’s knowledge about oral cancer, screening and attitudes toward screening, in addition to determining the barriers to oral cancer screening.

**Materials and Methods:**

A cross-sectional web-based study was conducted. The first section of the questionnaire employed collected the participants’ sociodemographic data. A question about whether patients had heard about oral cancer was then included, and those who answered ‘no’ were instructed to submit the questionnaire. The subsequent parts evaluated the participants’ knowledge of oral cancer and screening, attitudes toward screening, and barriers against screening. ANOVA and chi-squared tests were conducted to investigate the sample characteristics associated with the participants’ unfamiliarity with oral cancer. Binary regression was conducted to predict the variables associated with the participants’ knowledge and attitudes.

**Results:**

The questionnaire was filled by 1307 participants (1011 females). Most of the participants (70.01%) stated that they had heard of oral cancer, and the variables associated with awareness of oral were sex, monthly income, health insurance coverage, working status, and educational level. Sources of information and age were significantly associated with knowledge and attitude levels. The most ‘agree/strongly agree’ responses about barriers were lack of knowledge and lack of awareness.

**Conclusion:**

The study participants had moderate knowledge about oral cancer and satisfactory attitudes toward screening. Nearly all barriers to screening can be overcome by the joint efforts of healthcare providers and health authorities.

Oral cancer is one of the most problematic cancers. It is the sixth most common cause of cancer-related deaths in the world, with up to 400,000 new cases every year and almost 130,000 deaths annually.^22^ A few of the risk factors for oral cancer include older age, poor nutrition, and ultraviolet light exposure.^17^ However, the most important risk factor for oral cancer is tobacco smoking,^35^ which is the clear cause of many tumors in the oral cavity, lung, esophagus, and pancreas.^49^ Jordan has one of the highest rates in the world of smoking among adults, as nearly 30% of the population smoke tobacco,^18^ also in waterpipes, which is one of highest globally.^6,27,31^ These high rates mean that a high percentage of the Jordanian population is at risk of oral cancer.

Improving public knowledge about oral cancer risk factors, symptoms and methods of early detection may improve the attitudes and practices related to oral cancer, which in turn may decrease the prevalence rates of this disease. Moreover, as early detection of oral cancer can reduce mortality and improve treatment outcomes,^32^ improving public knowledge about oral cancer symptoms and methods of detection is essential. Studies conducted in the United Arab Emirates,^1^ Iraq,^4^ Saudi Arabia,^3^ and Sudan^20^ reported inadequate knowledge about oral cancer among their populations. In Jordan, few studies have investigated the knowledge, attitude, and practices toward oral cancer among different subgroups in the Jordanian population. The results of these studies indicated that awareness and knowledge about oral cancer in Jordan is inadequate in various population groups, including the general public, students and dental professionals.^2,26,37^ However, none of these studies addressed the knowledge about, attitude towards, and barriers to oral cancer screening. Therefore, the aims of this study were: to assess the knowledge about, attitude towards, and barriers to oral cancer screening among the Jordanian population; to explore the factors associated with each domain, identify knowledge gaps, and provide appropriate policy suggestions.

## Materials and Methods

The current cross-sectional study involved an online questionnaire that was distributed on generic Jordanian Facebook groups after obtaining ethical approval from the Institutional Review Board at Jordan University of Science and Technology. The inclusion criteria for study participants included being ≥18 years old and an Arabic-speaking resident of Jordan.

### Study Instrument and Questionnaire Development

After extensive literature review,^5,40,47^ the current study survey was developed. The survey began with a brief introduction describing study objectives and provided a simple definition of oral cancer. Furthermore, it emphasised the confidentiality of the participants. The participants were informed that completing the survey represented consent to participate in the study. The survey included four parts: part one contained information about sociodemographic variables including age, gender, marital status, smoking status, occupation, living conditions, income in Jordanian dinar (JD), educational level, area of residency (urban vs rural), specific area of residency (governate), family history and insurance status. The participants were also asked about knowing someone with cancer, if they had heard of cancer warning signs, if they had ever been screened for oral cancer, and the source of information they had received about oral cancer. Part two included 33 items that could be answered with ‘yes’ or ‘no’ to assess the knowledge of study participants. The information about oral cancer necessary to formulate part two of the questionnaire was retrieved from https://www.cancer.net/cancer-types/oral-and-oropharyngeal-cancer/symptoms-and-signs.^12^ The knowledge questions covered general knowledge about oral cancer (3 questions), risk factors for oral cancer (8 questions), symptoms of oral cancer (14 questions) and methods of early detection of oral cancer (8 questions). This 24-item knowledge questionnaire was adopted from the Alshammari et al^5^ study. The third part of the questionnaire was composed of six items to assess the attitude of study participants towards early screening of oral cancer. The attitude questionnaire was adopted from Wang et al.^47^ Part four was adopted from Alshammari et al^5^ and Muthukrishnan et al,^40^ and it included eleven items that explored barriers to oral cancer screening among the study participants. Participants answered part-3 and -4 questions on a five-point Likert scale from ‘strongly agree’ to ‘strongly disagree’. The 4-part structured survey was reviewed by experts in the field (family physician, dentists [including oral and maxillofacial surgeons], a professor of medicine, and an oncologist), and minor changes were implemented to produce the final version of the questionnaire. A pilot study was conducted on 20 individuals to test comprehensibility and to identify any difficulties with the questionnaire. The pilot data were excluded from the main study.

Participants’ main sources of information about oral cancer were divided into three groups: 1. reliable sources including physicians, TV, newspaper, radio, healthcare educational booklets, websites, and educational campaigns; 2. non-reliable source including friends and social media; 3. no-knowledge group.

The knowledge score was calculated based on the participants’ responses to part 2 of the questionnaire: a ‘yes’ response was granted 1 point; ‘no’ and ‘I don’t know’ responses were granted 0 points. The participants’ knowledge status was categorised as good or poor knowledge according to the median score; those who scored above the median had good knowledge and those who scored below the median had poor knowledge. The attitude score was computed based on the participants’ responses to part-3 questions. The points varied from 1 point for ‘strongly disagree’ and 5 points for ‘strongly agree’ for the items that evaluated the favourable attitudes toward early screening of oral cancer. For statements that evaluated unfavourable attitudes, a reversed scale was used. The participants were divided into two categories based on the calculated median of the attitude score.

### Statistical Analysis

Data analyses were conducted using SPSS version 27 (IBM; Armonk, NY, USA). The categorical variables were presented as frequencies and percentages, and the continuous variables were presented as means and standard deviations (SD). ANOVA and chi-squared tests were conducted to determine possible association of sample characteristics with the participants’ unfamiliarity with oral cancer. Binary regression was conducted to explore the variables associated with the participants’ knowledge and attitudes. p-values < 0.05 were considered statistically significant.

## Results

The datasets generated and/or analysed in the current study are available in the Mendeley repository, http://dx.doi.org/10.17632/v3gf5kzxhc.^1^

This population’s sociodemographics are shown in Table 1. A total of 1307 (296 males) participants completed the questionnaire. The mean age of the participants was 26.1 (± 9.8) years. The majority of the participants were single (76.1%) and nonsmokers (78.0%). The participants were divided almost equally between the two household-income/month groups (50.7%: < 1000 JD; 49.3%: ≥ 1000 JD). The majority of the sample had health insurance (66%) and more than 90% of the participants had a Bachelor’s degree or higher.

**Table 1:** Sociodemographic data collected

		Frequency (%) or mean (± SD)‏
Age		26.10 (9.77)
Sex	Female	1011 (77.35)
	Male	296 (22.66)
Marital status	Not married	1018 (77.9)
	Married	289 (22.11)
Smoking status	Nonsmoker	1068 (81.33)
	Smoker	244 (18.67)
Household average monthly income	Less than 1000 JD	663 (50.73)
	1000 or more JD	644 (49.27)
Health-insurance coverage‎	No	444 (33.97)
	Yes	863 (66.03)
Working status	Medical field	756 (57.84)
	Non-medical field	551 (42.16)
Educational level	Secondary school/highschool	130 (9.95)
	Bachelor’s degree or higher	1177 (90.05)

JD: Jordanian dinar.

As shown in Table 2, 30.0% of the participants had never heard of oral cancer. ANOVA and chi-squared tests identified several variables which were significantly associated with the recognition of oral cancer. The number of women who had heard about oral cancer was statistically significantly higher than the number of men (p < 0.001). The number of participants with a higher educational level and/or higher income who had heard or oral cancer was statistically significantly higher than those with a lower educational level and/or lower income (p < 0.001 and < 0.05, respectively). Lastly, the number of medical-field workers who had heard of oral cancer was statistically significantly higher than the number of non-medical-field workers (p < 0.001).

**Table 2:** Responses to the question ‘Have you heard about oral cancer?’

Variables		Frequency (%) or mean (± SD)	
		Have you ever heard about oral cancer?	
		Yes (n = 915, 70.0%)	No (n = 392, 30%)
Age		26.26 (± 9.61)	25.72 (± 10.15)
Sex	Female	731 (72.3)**	280 (27.7)
	Male	184 (62.2)	112 (37.8)
Marital status	Not married	717 (70.4)	301 (29.6)
	Married	198 (68.5)	91 (31.5)
Smoking status	Nonsmoker	753 (70.8)	310 (29.2)
	Smoker	162 (66.4)	82 (33.6)
Household average monthly income	Less than 1000 JD	449 (67.7)	214 (32.3)
	1000 or more JD	466 (72.4)*	178 (27.6)
Health insurance coverage	No	287 (64.6)	157 (35.4)
	Yes	628 (72.8)*	235 (27.2)
Working status	Medical field	602 (79.6)**	154 (20.4)
	Non-medical field	313 (56.8)	238 (43.2)
Educational level	Secondary school/highschool	63 (48.5)	67 (51.5)
	Bachelors’ degree or higher	852 (72.4)**	325 (27.6)

*Significant at p < 0.05; **significant at p < 0.001. JD: Jordanian dinar.

As shown in Table 3, about one-third (31.58%) of the participants had never heard about cancer warning signs. The main sources of participants’ information about oral cancer were websites (29.3%), social media (20.9%) and physicians (16.3%).

**Table 3:** History of cancer and screening

		Frequency (%)
Family history of oral cancer‎	I had oral cancer	1 (0.11)
	Someone in my family had oral cancer	20 (2.19)
	None of my family members had oral cancer	894 (97.70)
Family history of cancer other than oral cancer‎	I had cancer	11 (1.20)
	Someone in my family had cancer	312 (34.10)
	None of my family members had cancer	592 (64.70)
Do you know someone with cancer?	No	230 (25.14)
	Yes	685 (74.86)
Have you heard of cancer warning signs?	No	289 (31.58)
	Yes	626 (68.42)
Have you ever undergone screening for oral cancer?	No	902 (98.58)
	Yes	13 (1.42)
What is your main source of information about oral cancer?	Friends	46 (5.03)
	Physicians	149 (16.28)
	TV	28 (3.06)
	Newspaper	1 (0.11)
	Radio	2 (0.22)
	Health educational booklets	53 (5.79)
	Websites	268 (29.29)
	Educational campaigns	53 (5.79)
	I have no knowledge about oral cancer	124 (13.55)
	Social media	191 (20.87)

Table 4 represents the participants’ responses to the questions that assessed their knowledge about oral cancer. The most known risk factor for oral cancer was smoking (92.8%), followed by alcohol consumption (83.3%), while prolonged sun exposure was the least known risk factor (20.7%) among the study participants. Regarding oral cancer symptoms, all symptoms were known by at least 50% of the participants. More than 80% of the participants recognised lump on the lip, mouth or throat, and difficulty chewing, swallowing, or moving the jaws or tongue as symptoms of oral cancer. The least known symptom was changes of speech (52.0%). Regarding methods of early detection of oral cancer, more than 90% of the participants acknowledged regular checkup for physical examination as a method of early detection. The second most known method was oral brush biopsy (75.1%), while the least known method was barium swallow/modified barium swallow (27.5%). The knowledge score median was 22 out of 33.

**Table 4:** Participants’ knowledge about oral cancer (general knowledge, risk factors, symptoms, methods for screening)

	No	I don’t know	Yes
Have you ever heard of early cancer testing?	234 (25.57)	-	681 (74.43)
I think oral cancer is fatal	111 (12.13)	212 (23.17)	592 (64.7)
Oral cancer recovery rates increase when detected in the early stages	10 (1.09)	98 (10.71)	807 (88.2)
**Risk factors for oral cancer**			
Prolonged sun exposure	481 (52.57)	245 (26.78)	189 (20.66)
Infection with HPV	82 (8.96)	243 (26.56)	590 (64.48)
Poor oral hygiene	160 (17.49)	133 (14.54)	622 (67.98)
Alcohol consumption	66 (7.21)	87 (9.51)	762 (83.28)
Increasing age	289 (31.58)	187 (20.44)	439 (47.98)
Smoking	22 (2.4)	44 (4.81)	849 (92.79)
Poor diet	227 (24.81)	174 (19.02)	514 (56.17)
The risk increased based on the individual’s sex	274 (29.95)	307 (33.55)	334 (36.5)
**Symptoms of oral cancer**			
Sore in the mouth or on the lip that does not heal	57 (6.23)	161 (17.6)	697 (76.17)
Red or white patch on the gums, tongue or lining of the mouth	136 (14.86)	186 (20.33)	593 (64.81)
Lump on the lip, mouth or throat	31 (3.39)	138 (15.08)	746 (81.53)
Lump on the neck	177 (19.34)	242 (26.45)	496 (54.21)
Persistent sore throat or feeling something caught in your throat	81 (8.85)	204 (22.3)	630 (68.85)
Numbness in the mouth, lips or tongue	104 (11.37)	255 (27.87)	556 (60.77)
Hoarseness or change in voice	95 (10.38)	184 (20.11)	636 (69.51)
Changes of speech	179 (19.56)	260 (28.42)	476 (52.02)
Difficulty chewing, swallowing, or moving the jaws or tongue	40 (4.37)	135 (14.75)	740 (80.87)
Pain or bleeding in the mouth	50 (5.46)	179 (19.56)	686 (74.97)
Loosening of teeth	116 (12.68)	253 (27.65)	546 (59.67)
Ear and/or jaw pain	112 (12.24)	257 (28.09)	546 (59.67)
Unexplained weight loss	114 (12.46)	202 (22.08)	599 (65.46)
Chronic bad breath	100 (10.93)	215 (23.5)	600 (65.57)
**Methods of early detection of oral cancer**			
Regular checkup for physical examination	18 (1.97)	44 (4.81)	853 (93.22)
Endoscopy	176 (19.23)	193 (21.09)	546 (59.67)
Oral brush biopsy	88 (9.62)	140 (15.3)	687 (75.08)
Barium swallow/modified barium swallow	239 (26.12)	424 (46.34)	252 (27.54)
Blood tests	143 (15.63)	163 (17.81)	609 (66.56)
Panoramic radiographs	285 (31.15)	308 (33.66)	322 (35.19)
CT scan	106 (11.58)	255 (27.87)	554 (60.55)
MRI	74 (8.09)	176 (19.23)	665 (72.68)

Table 5 displays the participants’ attitudes toward early screening of oral cancer and the barriers to oral cancer screening. The attitude score median was 27 out of 30. More than 90% of the participants agreed or strongly agreed with the following statements ‘It is important for me to know about cancer’, ‘Cancer screening should be implemented on a large scale’, and ‘If oral cancer is diagnosed at an early stage, the treatment outcomes can be better’.

**Table 5:** The participants’ opinions regarding early screening of oral cancer and the barriers to early screening

Question	Strongly disagree	Disagree	Neutral	Agree	Strongly agree
**Assessment of attitude towards early screening of oral cancer**					
It is important for me to know about cancer	0 (0)	6 (0.7)	35 (3.8)	240 (26.2)	634 (69.3)
Someone who has cancer is simply unlucky	168 (18.4)	373 (40.8)	209 (22.8)	81 (8.9)	84 (9.2)
Cancer screening should be implemented on a large scale	1 (0.1)	9(1)	57 (6.2)	248 (27.1)	600 (65.6)
I believe that regular physical examinations help to detect oral cancer in early stages	0 (0)	11(1.2)	51(5.6)	222 (24.3)	631 (69)
If oral cancer is diagnosed in early stage, the treatment outcomes can be better	0 (0)	9(1)	46(5)	211 (23.1)	649 (70.9)
Smoking cessation is important to prevent oral cancer	0 (0)	9 (1)	75 (8.2)	161 (17.6)	670 (73.2)
**Assessment of barriers to oral cancer screening**					
Lack of knowledge about oral cancer	0 (0)	12 (1.3)	48 (5.2)	333 (36.4)	522 (57)
Lack of awareness of the need for the screening	1 (0.1)	11 (1.2)	48 (5.2)	328 (35.8)	527 (57.6)
Having no symptoms	4 (0.4)	37 (4)	174 (19)	334 (36.5)	366 (40)
Anxiety of screening procedure	3 (0.3)	59 (6.4)	146 (16)	346 (37.8)	361 (39.5)
Fear of finding cancer	8 (0.9)	50 (5.5)	99 (10.8)	308 (33.7)	450 (49.2)
Young age	8 (0.9)	51 (5.6)	196 (21.4)	339 (37)	321 (35.1)
Lack of time	33 (3.6)	149 (16.3)	241 (26.3)	251 (27.4)	241 (26.3)
Difficulty of getting an appointment for the screening	28 (3.1)	131 (14.3)	250 (27.3)	246 (26.9)	260 (28.4)
Difficulties in transportation	38 (4.2)	185 (20.2)	268 (29.3)	204 (22.3)	220 (24)
Cost or lack of insurance	7 (0.8)	39 (4.3)	108 (11.8)	328 (35.8)	433 (47.3)
Lack of professional healthcare recommendations	3 (0.3)	30 (3.3)	104 (11.4)	328 (35.8)	450 (49.2)

Most of the participants (> 90%) agreed or strongly agreed that lack of knowledge about oral cancer and lack of awareness of the need for screening are barriers to oral cancer screening. On the other hand, ‘difficulties in transportation’ was the least reported barrier to screening.

Binary regressions were conducted to determine the predictors for being in different knowledge and attitude groups. As shown in Table 6, working in the medical field (B = 1.72, p < 0.001) and increased age (B = 0.03, p = 0.01) statistically significantly increased the odds of being in the high-knowledge group On the other hand, having no knowledge about oral cancer or relying on non-reliable sources for information about oral cancer (B = -0.37, p = 0.023) and increased age (B = -0.02, p = 0.30) statistically significantly decreased the odds of being in the favourable-attitude group.

**Table 6:** Binary regression of knowledge score

Variable	B	p-value	95% CI for OR	
			Lower	Upper
Sex Females vs males	0.16	0.419	0.79	1.76
Educational level Secondary school/highschool vs Bachelor’s degree or higher	-0.36	0.263	0.37	1.31
Residency area Rural vs urban	0.30	0.096	0.95	1.93
Working field Medical field vs non-medical field	1.72	<0.001*	3.85	8.06
Marital status Not married vs married	0.07	0.775	0.66	1.74
Smoking status Nonsmokers vs smokers	-0.20	0.349	0.54	1.25
Household average monthly income Less than 1000 JD vs 1000 JD or more	-0.21	0.163	0.61	1.09
Do you know someone with cancer? No vs yes	0.10	0.540	0.80	1.54
What is your main source of information about oral cancer? ‘Non-reliable sources’ and ‘reliable sources’ groups vs ‘I have no knowledge’ group	-0.75	0.004*	0.28	0.79
What is your main source of information about oral cancer? ‘Non-reliable’ and ‘I have no knowledge’ groups vs ‘reliable sources’ group	-0.18	0.300	0.60	1.17
Age	0.03	0.010*	1.01	1.05

*Statistically significant. JD: Jordanian dinar.

**Table 7:** Binary regression of attitude score

	B	p-value	95% CI for OR	
			Lower	Upper
Sex Females vs males	0.22	0.261	0.85	1.80
Educational level Secondary school/highschool vs Bachelor’s degree or higher	-0.61	0.042	0.30	0.98
Residency area Rural vs urban	-0.07	0.680	0.66	1.31
Working field Medical field vs nonmedical field	-0.15	0.382	0.61	1.21
Marital status Not married vs married	0.07	0.775	0.67	1.71
Smoking status Nonsmokers vs smokers	0.22	0.264	0.85	1.85
Household average monthly income Less than 1000 JD vs 1000 JD or more	-0.15	0.283	0.65	1.13
Do you have health insurance? No vs yes	-0.20	0.198	0.67	1.11
Do you know someone with cancer? No vs yes	-0.09	0.561	0.61	1.24
What is your main source of information about oral cancer? ‘Non-reliable sources’ and ‘feliable sources’ groups vs ‘I have no knowledge’ group	0.12	0.609	0.71	1.79
What is your main source of information about oral cancer? ‘Non-reliable’ and ‘I have no knowledge’ groups vs ‘reliable sources’ group	-0.37	0.023*	0.50	0.95
Age	-0.02	0.030*	0.96	1.00
Knowledge score	0.02	0.085	1.00	1.04

*Statistically significant. JD: Jordanian dinar.

## Discussion

In 2012, the incidence rate of oral cancer in Jordan was 1.7 per 100,000 population, with a mortality rate of 0.6 per 100,000.^34^ Research has shown that early detection of oral cancer increases the survival rate and improves the disease outcome. To improve the rates of early detection of cancer, two strategies were suggested by Early Detection Knowledge into Action Cancer Control, WHO Guide for Effective Programmes.^16^ The first strategy includes early diagnosis, involving increasing patients’ awareness of early signs and symptoms, which prompts the patient to consult a healthcare provider. The second strategy comprises national screening of asymptomatic patients.^16^ In order to put these strategies in action, the gaps in knowledge and attitudes toward oral cancer as wel as the barriers to screening should be identified and targeted in future health care programs for oral cancer patients.

### Knowledge about Oral Cancer

Two-thirds of this study’s participants had heard about oral cancer, while another study that evaluated the knowledge about oral cancer in Jordan reported less than 50% of dental outpatients had done so.^26^ On the other hand, a higher percentage (90%) of those who have heard about oral cancer was reported by a study including the University of Jordan students.^37^ The higher percentage among the students is in line with the current study’s results, as the number of participants with at least a Bachelors’ degree who acknowledged having heard about oral cancer was statistically significantly higher vs the participants with an educational level of at most secondary school. Similarly, the percentage of participants who work in the medical field and had never heard of oral cancer was statistically significantly lower than the percentage observed among workers not in the medical field. Moreover, those who have ever heard about oral cancer among workers not in the medical field had statistically significantly lower knowledge scores compared to medical-field workers. These results warrant simple and easily understandable educational campaigns targeted toward non-medical-field workers and those with low educational levels. Targeting the low socioecononic-status (SES) population is also of a particular importance, as studies have shown that this population may have higher cancer incidence rates and increased rates of cancer diagnosed at a late stage.^15,48^ Besides, tobacco smoking prevalence among low SES groups is statistically significantly higher than the prevalence among those with high SES,^23^ which may expose this group to a higher risk of oral cancer.

Sex was also a contributor to the participants’ knowledge about oral cancer: a statistically significantly lower percentage of males acknowledged that they have heard about oral cancer. This result should be a cause of concern for Jordanian health authorities, as the number of males in Jordan who smoke is high,^10^ which may in turn increase the risk of oral cancer.

Although physicians are one of the most reliable sources of information about oral cancer, only 16.3% of the participants acknowledged them as the main source of information about oral cancer. Training programs for physicians, particularly dentists, should be implemented to improve their knowledge about oral cancer and hence improve their participation in patient education about the disease.

### Oral Cancer Risk Factor

In line with several other studies,^25,28,44^ tobacco smoking was identified as a risk factor for oral cancer by most of this study’s participants. Previous studies among different healthcare professions reported comparable results, indicating that the general population of Jordan has proper knowledge of the dangers of smoking.^30,33^ This result should encourage healthcare professionals, particularly dentists, to counsel their patients about smoking cessation programs.

In contrast, several other oral-cancer risk factors were not identified by more than half of the participants, including prolonged sun exposure and increasing age. These factors are especially important because they are associated with an increased risk of types of cancer other than oral, which confirms the great importance of being awareness of these factors.^8,50^

### Oral Cancer Symptoms

There were variations in the level of knowledge about oral cancer symptoms, ranging from 81% for lump on the lip, mouth, or throat to 52% for hoarseness or change in voice. This variation was also reported in earlier studies. For instance, in a study conducted in Iran, the level of the participants’ knowledge about oral cancer symptoms varied between 63.1% for chronically non-healing ulcers and 11.9% for bleeding.^7^ In another study conducted in Portugal, the participants’ level of knowledge regarding different oral cancer symptoms varied between 90% and 27.1%.^39^

The most common symptom of oral cancer, a sore in the mouth or on the lip that does not heal,^12^ was known to the majority of the participants. In line with an Indian study that targeted secondary school students,^45^ difficulty chewing, swallowing, or moving the jaws or tongue was one of the most well-known oral cancer symptoms amongst the current study participants. However, because oral cancer symptoms may vary depending on the spot in the mouth where cancer first develops,^38^ it is essential to be aware of all disease symptoms.

### Methods of Early Detection of Oral Cancer

Regular checkups (physical examinations) were known to the majority of the participants as one method of early detection of oral cancer.^9^ In practice, this is the most commonly used method because of its high accessibility. However, many of the oral cancer symptoms may be misclassified as a symptom of other, less serious conditions.^41^ Therefore, methods other than physical examination would be necessary if oral cancer were suspected. The participants’ familiarity with the other methods of early detection varied statistically significantly, as less than 30% were familiar with barium swallow/modified barium swallow. Familiarity with different methods of early detection of oral cancer may give the patients more confidence in the different methods and increase their willingness to make use of these methods. This hypothesis regarding the awareness of different oral cancer screening methods and their utilisation has yet to be tested; however, studies about screening methods for other types of cancer found a statistically significantly positive association between the awareness of a certain screening method and its utilisation.^19,24,42^ Also, a statistically significantly positive correlation was found between the participants’ awareness of a certain screening method and their attitudes toward its utilisation.^36^

### Attitudes toward Early Screening for Oral Cancer

Favourable attitudes toward early screening for oral cancer were observed amongst the participants, as the median of the attitudes score was 27 out of 30. The positive attitudes toward oral cancer screening in the current study are in line with the results of a study conducted in London.^11^

Older age is a risk factor for oral cancer, with the majority of oral cancer cases being diagnosed in patients over 40 years of age.^46^ In the current study, older age was associated with a better knowledge level but a negative attitude toward oral cancer screening. This could be attributed to the increased negative emotions such as fear, anxiety, embarrassment, pain and discomfort amongst the elderly.^14^ Hence, dentists should focus on older people when conducting oral cancer screening and consulting patients on the early symptoms of oral cancer and the importance of screening.

### Barriers to Conducting Oral Cancer Screening

Lack of knowledge about oral cancer was one of the most common barriers to oral cancer screening in the literature,^29,43,51^ which is consistent with the current study’s finding. In an earlier focus-group study that evaluated the barriers to oral cancer screening among rural Black American adults, the barrier ‘lack of knowledge’ accounted for 31.8% of all mentioned barriers.^29^ Likewise, in an article that reported the results of a focus-group discussion with irregular dental attenders at dental clinics in England, a striking lack of knowledge about oral cancer was reported; the authors suggested that increasing knowledge about oral cancer may improve the public’s use of oral cancer screening.^51^ Similarly, lack of awareness is another barrier that confronted the participants of the current study.

The majority of the present study’s participants agreed or strongly agreed that lack of professional healthcare recommendations is one of the barriers to oral cancer screening. In a study that analysed the effect of removing this barrier on participants’ intentions to be screened, removing the barrier ‘lack of professional healthcare recommendations’ had the largest effect on increasing screening.^43^

In the current study, difficulty in transportation was the least recognised barrier. A similar finding was observed in the previously mentioned Black American adult focus-group study, where this barrier was at the bottom of the barrier list.^29^

In general, most of the barriers identified in this study are within the healthcare providers’ and health authorities’ responsibilities, as increasing knowledge and awareness about oral cancer and removing cost as a barrier to screening cannot be done without the active involvement of these parties. Furthermore, patient-related barriers such as anxiety about screening procedures and fear of finding cancer can be surmounted by consultation with healthcare providers.

### Study Limitations and Strengths

The study results were based on data collected via an online questionnaire, which may result in recall and selection biases. However, research has proven that web-based studies can be used to recruit a representative sample and they provide a private environment which allows respondents to complete the questionnaire accurately and honestly.^13,21^ The distribution of the participants across medical vs non-medical fields is another limitation of the study, as more than half of the participants worked in a medical field, which may limit the generalisability of the results. However, knowledge and attitudes of the medical staff are particularly important, as they are the source of reliable information about oral cancer, and they must be included in future education and screening campaigns. Moreover, the study included a large sample size, which could decrease the influence of this limitation.

## Conclusion

This is the first study to assess knowledge, attitudes, and barriers to oral cancer screening in a Jordanian population. The current study’s participants did not demonstrate an optimum knowledge level of oral cancer screening. However, they showed positive attitudes toward this practice. Education campaigns that use simple, easily understood language should be implemented to improve knowledge and awareness about oral cancer screening practices, particularly for those with a high risk of developing oral cancer.

## References

[ref1] Al-Rawi N, Kawas S, Imad O (2012). Public awareness and attitude toward oral cancer screening In United Arab Emirates. J Int Dent Med Res.

[ref2] Alami AY, El Sabbagh RF, Hamdan A (2013). Knowledge of oral cancer among recently graduated medical and dental professionals in Amman, Jordan. J Dent Educ.

[ref3] Alqahtani M, Nahhas A, Malibari L, Alghamdi M, Bazuhier S, Abdulrahman S (2020). Awareness of oral cancer among dental patients in Mecca, Saudi Arabia. Open Dent J.

[ref4] Alshami M, Abdulbaqi H, Abdulkareem A (2020). Awareness and knowledge of oral cancer in the city of Baghdad, Iraq: a questionnaire-based survey. J Stomatol.

[ref5] Alshammari S, Alenazi H, Alshammari H (2020). Knowledge, attitude and practice towards early screening of colorectal cancer in Riyadh. J Fam Med Prim Care.

[ref6] Alzyoud S, Weglicki LS, Kheirallah KA, Haddad L, Alhawamdeh KA (2013). Waterpipe smoking among middle and high school Jordanian students: patterns and predictors. Int J Environ Res Public Health.

[ref7] Amanpour S, Raoof M, Kakoei S, Fardisi S, Iranmanesh A, Parizi MT (2018). Knowledge of Adults’ Reading Oral Cancer in South-East of Iran. Int J Epidemiol Res.

[ref8] Ananthaswamy HN (2001). Sunlight and skin cancer. J Biomed Biotechnol.

[ref9] Awan K (2014). Oral cancer: early detection is crucial. J Int Iral Health.

[ref10] Awidi AS (1991). Patterns of cigarette smoking in Jordan: a study of greater Amman area. Ann Saudi Med.

[ref11] Awojobi O, Scott SE, Newton T (2012). Patients’ perceptions of oral cancer screening in dental practice: a cross-sectional study. BMC Oral Heal.

[ref12] Cancer.Net https://www.cancer.net/cancer-types/oral-and-oropharyngeal-cancer/symptoms-and-signs.

[ref13] Cantrell MA, Lupinacci P (2007). Methodological issues in online data collection. J Adv Nurs.

[ref14] Chien S-Y, Chuang M-C, Chen I-P (2020). Why people do not attend health screenings: factors that influence willingness to participate in health screenings for chronic diseases. Int J Environ Res Public Health.

[ref15] Clegg LX, Reichman M, Hankey BF, Singh GK, Lin YD E, Miller BA (2009). Impact of socioeconomic status on cancer incidence and stage at diagnosis: Selected findings from the surveillance, epidemiology, and end results: National Longitudinal Mortality Study. Cancer Causes Control.

[ref16] Com Sàrl I (2007). Early detection knowledge into action cancer control.

[ref17] CTCA. Cancer Treatment Centers of America Top Oral Cancer Causes and Factors That Put You at Risk. cancercenter.com/cancer-types/oral-cancer/risk-factors.

[ref18] Dar-Odeh NS, Bakri FG, Al-Omiri MK, Al-Mashni HM, Eimar HA, Khraisat AS (2010). Narghile (water pipe) smoking among university students in Jordan: Prevalence, pattern and beliefs. Harm Reduct J.

[ref19] Elobaid YE, Aw TC, Grivna M, Nagelkerke N (2014). Breast cancer screening awareness, knowledge, and practice among Arab women in the United Arab Emirates: a cross-sectional survey. PLoS One.

[ref20] Eltayeb AS, Satti AE, Suleiman AM (2017). Oral cancer awareness in Sudan: assessment of knowledge, attitude and treatment seeking behaviour. Int J Oral Maxillofac Surg.

[ref21] Fenner Y, Garland SM, Moore EE, Jayasinghe Y, Fletcher A, Tabrizi SN (2012). Web-based recruiting for health research using a social networking site: An exploratory study. J Med Internet Res.

[ref22] Ferlay J, Shin HR, Bray F, Forman D, Mathers C, Parkin DM (2010). Estimates of worldwide burden of cancer in 2008: GLOBOCAN 2008. Int J Cancer.

[ref23] Garrett BE, Martell BN, Caraballo RS, King BA (2019). Socioeconomic differences in cigarette smoking among sociodemographic groups. Prev Chronic Dis.

[ref24] George TJ (2021). Factors influencing utilization of cervical cancer screening services among women –A cross sectional survey. Clin Epidemiol Glob Health.

[ref25] Halawany H, Jacob V, Abraham N, Al-Maflehi N (2013). Oral cancer awareness and perception of tobacco use cessation counseling among dental students in four Asian countries. Asian Pac J Cancer Prev.

[ref26] Hassona Y, Scully C, Abu Ghosh M, Khoury Z, Jarrar S, Sawair F (2015). Mouth cancer awareness and beliefs among dental patients. Int Dent J.

[ref27] Hawash M, Mosleh R, Hanani A, Jarar Y, Hajyousef Y A comparison of shisha smoking among university students in Palestine, Jordan and Turkey [Epub ahead of print]. https://assets.researchsquare.com/files/rs-19928/v1/5277cd62-ea6a-49c2-b26e-2fe87ed1d2fc.pdf?c=1631832483.

[ref28] Hertrampf K, Wenz HJ, Koller M, Wiltfang J (2012). Public awareness about prevention and early detection of oral cancer: A population-based study in Northern Germany. J Cranio-Maxillofacial Surg.

[ref29] Howell JL, Shepperd JA, Logan H (2013). Barriers to oral cancer screening: A focus group study of rural Black American adults. Psychooncol.

[ref30] Jaber L, Shaban S, Hariri D, Smith S (2011). Perceptions of healthcare practitioners in Saudi Arabia regarding their training in oral cancer prevention, and early detection. Int J Health Care Qual Assur.

[ref31] Jaghbir M, Shreif S, Ahram M (2014). Pattern of cigarette and waterpipe smoking in the adult population of Jordan. East Mediterr Health J.

[ref32] Jitender S, Sarika G, Varada HR, Omprakash Y, Mohsin K (2016). Screening for oral cancer. J Exp Ther Oncol.

[ref33] Joseph BK, Sundaram DB, Sharma P (2012). Oral cancer awareness among dentists in Kuwait. Med Princ Pract.

[ref34] Kujan O, Farah CS, Johnson NW (2017). Oral and oropharyngeal cancer in the Middle East and North Africa. Transl Res Oral Oncol.

[ref35] Kumar M, Nanavati R, Modi TG, Dobariya C (2016). Oral cancer: Etiology and risk factors: A review. J Cancer Res Ther.

[ref36] Manzour AF, Gamal Eldin DA (2019). Awareness about breast cancer and mammogram among women attending outpatient clinics, Ain Shams University Hospitals. Egypt. J Egypt Public Heal Assoc.

[ref37] Masadeh M (2018). Students’ knowledge regarding oral cancer at the University of Jordan: a preliminary study. J Soc Sci.

[ref38] Moffitt Cancer Center What Are the First Signs of Oral Cancer?. https://moffitt.org/cancers/oral-cavity-or-throat-cancer/faqs/what-are-the-first-signs-of-oral-cancer/.

[ref39] Monteiro LS, Salazar F, Pacheco J, Warnakulasuriya S (2012). Oral cancer awareness and knowledge in the city of Valongo, Portugal. Int J Dent.

[ref40] Muthukrishnan M, Arnold LD, James AS (2019). Patients’ self-reported barriers to colon cancer screening in federally qualified health center settings. Prev Med Reports.

[ref41] Scully C, Newman L, Bagan JV (2005). The role of the dental team in preventing and diagnosing cancer: 3. oral cancer diagnosis and screening. Dent Update.

[ref42] Shankar A, Roy S, Rath GK, Chakraborty A, Kamal VK, Biswas AS (2018). Impact of cancer awareness drive on generating awareness of and improving screening for cervical cancer: a study among schoolteachers in India. J Glob Oncol.

[ref43] Shepperd JA, Howell JL, Logan H (2014). A survey of barriers to screening for oral cancer among rural Black Americans. Psychooncol.

[ref44] Shimpi N, Jethwani M, Bharatkumar A, Chyou PH, Glurich I, Acharya A (2018). Patient awareness/knowledge towards oral cancer: A cross-sectional survey. BMC Oral Health.

[ref45] Talib M, Gupta P, Bhardwaj P (2018). Knowledge, attitude and practices regarding oral cancers amongst secondary school students in Lucknow district. Int J Community Med Public Health.

[ref46] The Oral Cancer Foundation Oral Cancer Facts. https://oralcancerfoundation.org/facts/.

[ref47] Wang MY, Lin GZ, Li Y, Dong H, Liao YH, Liu HZ (2017). Knowledge, attitudes, preventive practices and screening intention about colorectal cancer and the related factors among residents in Guangzhou, China. Asian Pacific J Cancer Prev.

[ref48] Wang N, Cao F, Liu F, Jia Y, Wang J, Bao C (2015). The effect of socioeconomic status on health-care delay and treatment of esophageal cancer. J Transl Med.

[ref49] Warnakulasuriya KAAS, Johnson NW, Linklater KM, Bell J (1999). Cancer of mouth, pharynx and nasopharynx in Asian and Chinese immigrants resident in Thames regions. Oral Oncol.

[ref50] White MC, Holman DM, Boehm JE, Peipins LA, Grossman M, Jane Henley S (2014). Age and cancer risk: A potentially modifiable relationship. Am J Prev Med.

[ref51] Zohoori FV, Shah K, Mason J, Shucksmith J (2012). Identifying factors to improve oral cancer screening uptake: a qualitative study. PLoS One.

